# Aging and cancer: Is glucose a mediator between them?

**DOI:** 10.18632/oncotarget.27344

**Published:** 2019-11-26

**Authors:** Alexey G. Golubev, Vladimir N. Anisimov

**Affiliations:** ^1^Department of Carcinogenesis and Oncogerontology, N.N. Petrov National Medical Research Center of Oncology, Saint Petersburg 197758, Russia

**Keywords:** cancer, aging, risk factor, glucose, lipid metabolism

## Abstract

Aging can increase cancer incidence because of accumulated mutations that initiate cancer and via compromised body control of premalignant lesions development into cancer. Relative contributions of these two factors are debated. Recent evidence suggests that the latter is rate limiting. In particular, hyperglycemia caused by compromised body control of blood glucose may be a factor of selection of somatic mutation-bearing cells for the ability to use glucose for proliferation. High glucose utilization in aerobic glycolysis is a long known characteristic of cancer. The new evidence adds to the concepts that have been being developed starting from mid-1970ies to suggest that age-related shifts in glucose and lipid metabolism increase the risk of cancer and compromise prognoses for cancer patients and to propose antidiabetic biguanides, including metformin, for cancer prevention and as an adjuvant means of cancer treatment aimed at the metabolic rehabilitation of patients. The new evidence is consistent with several effects of glucose contributing to aging and acting synergistically to enhance carcinogenesis. Glucose can affect (i) separate cells (via promoting somatic mutagenesis and epigenetic instability), (ii) cell populations (via being a factor of selection of phenotypic variants in cell populations for higher glucose consumption and, ultimately, for high aerobic glycolysis); (iii) cell microenvironment (via modification of extracellular matrix proteins), and (iv) the systemic levels (via shifting the endocrine regulation of metabolism toward increasing blood lipids and body fat, which compromise immunological surveillance and promote inflammation). Thus, maintenance of youthful metabolic characteristics must be important for cancer prevention and treatment.

## Introduction

Relationships between aging and cancer is a focus of longstanding attention in biomedical research [1–6]. A consensus is that age is indeed a major cancer risk factor. However, the way(s) of translation of (biological) time/age into cancer risk is/are debated.

Several principal approaches to the problem may be delineated. One is to think that cancer incidence (and, thus, cancer-related death rate) increases with age by its own, e.g., due to the accumulation of a specific kind of mutations, whereas aging (and the resulting increase in the total mortality rate) develops by its own pathways and does not interfere with carcinogenesis, so that age-dependent increases in the rates of deaths attributable to cancer and to all other causes are merely correlated more or less via the physical time. This attitude is famously proclaimed by R. Peto: “There is no such thing as ageing, and cancer is not related to it” [7]. Indeed, in human populations, which due to their sizes provide the most reliable data about relationships between mortality related to cancer and to other causes, mortality rates attributable to specific cancers increase with age by various trajectories, which are mostly different from the trajectory of total mortality [4]. This might suggest that to combat cancer is to control the specific causes of each cancer type. However, the combined mortality from all cancers increases in a way parallel to that of the total mortality, which in the middle age span is best described with an exponent (the Gompertz law) [4, 8]. This parallelism suggests three other approaches, which are not mutually excluding. Assuming that carcinogenesis and aging may share some common pathways, one might think that slowing down aging by targeting such pathway(s) will attenuate cancer initiation or/and progression, whereas targeting the related pathways of carcinogenesis will slow down aging. Or else assuming that aging attenuates the resistance of (human) body to any causes of death, one may suggest that these causes include cancer, i.e., aging attenuates the ability of organism to counteract carcinogenesis, e.g., because cell immunity and/or the ability of normal tissue cells to compete with transformed cells for resources available to a tissue become increasingly compromised in the course of aging. Another assumption is that aging makes body increasingly conducive to cancer. The latter two assumptions are opposite in that one implies that aging attenuates body resistance to cancer (– × – = +), whereas the other that aging potentiates carcinogenesis (+ × + = +). However, both of the assumption imply that slowing down of aging will decelerate an increase in the total cancer-related death rate.

The last two cases are supported, in particular, in a series of publications by J. DeGregori and coauthors [9–12] who provided compelling evidence that the age-dependent accumulation of mutations plays a relatively minor role in the increased incidence of cancer with age. The authors stressed that the mutation-centric theory is unable to explain numerous phenomena, such as the disproportion between cancer frequency and animal body size and the scaling of cancer incidence with animal lifespan, and suggested that cancer is rate limited by physiological aging-dependent factors.

### Glucose as a factor of clonal selection for malignant traits

The issue of what are the particular cancer-limiting physiological aging-dependent processes has been addressed recently in the paper [13] where its authors pointed out, in particular, that “…surveys of public health trends predict that smoking as the leading modifiable cause of cancer will soon be overtaken by metabolic-imbalance, caused by overconsumption of calories and lack of physical activity and with the obvious clinical sequelae of obesity and type 2 diabetes… In obesity and diabetes, the availability of metabolites, especially carbohydrates, fatty acids and lipids, are altered; the activity of many metabolic regulators is also perturbed, including insulin and IGFs, and obesity is associated with a chronic low inflammatory state and the levels of many inflammatory cytokines are also affected… In tumors with considerable heterogeneity these changes in the internal environment will alter the clonal selection pressures, and clones that can take advantage of the increased supply of energy, nutrients and stimulants will gain an advantage”.

A noteworthy elaboration of these lines of thinking may be found in the recent paper where “energy oversupply to tissues” was suggested to be “a single mechanism possibly underlying multiple cancer risk factors” [14]. The authors proposed “*the metabolic cancer suppression* hypothesis as a potential mechanistic explanation for the association between cancer risk and oversupply of energy to tissues”. The hypothesis posits that dysregulated oversupply of energetic resources to somatic cells abrogates the normal metabolic cancer suppression created through organismal limitation of tissue energy supply, and thereby accelerates cellular evolution toward cancer. Based on evidence that the risk of several types of cancer is significantly associated with diabetes and hyperglycemia, the authors reason that this association appears to be causal, with diabetes and hyperglycemia increasing cancer mortality across multiple cancer types. “Obesity appears to arise from, but not contribute to, this causal chain, as hyperglycemia is associated with cancer risk for several organ sites independently of obesity… The most direct cancer risk factor associated with obesity is a history of chronic hyperglycemia… Indeed, experimental evidence from an animal model indicates that their association arises because obesity and cancer both arise from the same upstream causal factors, including a history of chronic positive energy balance and chronic hyperglycemia”. The authors supported their hypothesis with agent-based modeling to show that excess energy availability can accelerate carcinogenesis via increased cell proliferation, which may be oncogenic because it modifies selective pressures, creating positive selection for oncogenic cell traits that in normal tissue are weakly, or even negatively, selected. “The hypothesis predicts that the effect of physical activity most relevant to cancer prevention may be reduction of chronic hyperglycemia” [14].

### Everything new is actually well overlooked old

Taken together, these sprouts of novelty are combined into what may seem a revelation; however, not to those who for decades have been cultivating the soil for the sprouts to burst.

Here is a quotation from a 30 years-old review [15] of studies carried out still earlier: “Thus, the same metabolic shifts promote the proliferation of nonlymphoid cells of certain types on the one hand and, on the other, they inhibit the cellular immunity, i.e., they induce the state of cancrophilia - a complex of metabolic conditions promoting cancer development. In favor of the role of cancrophilia in cancer development, the following experimental and clinical evidence may be cited: (1) treatment of C3H mice with phenformin reduces the cumulative incidence of mammary carcinomas down to 20% in an experimental group, compared with 80% in the control group (Dilman and Anisimov, 1980); 2) phenformin reduces the DMBA-induced mammary cancer incidence in rats (Dilman et al., 1978); (3) calorie restriction improves the indices of cellular immunity in experimental animals and leads to prolongating the mean life-span (Fernandez et al., 1978; Weindruch et al., 1982); (4) numerous observations in the human populations show that excessive consumption of dietary fat and cholesterol and excessive calories in general correlate with the increased incidence of mammary, endometrial, prostate, colon cancers, and tumors of some other localizations”.

The effects of antidiabetic biguanides, including phenformin, are manifested in improving body tolerance to glucose and thus in reducing the exposure of cells to glucose. Although phenformin is currently substituted for with metformin as an agent believed to be able to contribute to cancer prevention and treatment [16–18], both drugs fit the concept of metabolic rehabilitation of cancer patients, which was advocated more than 30 years ago (see [19]). Antidiabetic biguanides are not mentioned in [14]; however, it is highly unlikely that the authors who talk about hyperglycemia as a factor of carcinogenesis are unaware of the anticancer effects of antidiabetic glucose-lowering drugs and of the origin of the idea that they must have such effects.

Now what about the ability of cancer cells to benefit from an increase in glucose availability? Here is an almost 40 years-old paper by V. Dilman and M. Blagosklonny [20]: “It is suggested that the transforming protein (type pp60) induces “insulinization”…, which in turn results in the increased glucose transport into cell … sufficient to explain “the biochemical behaviour” of the cancerous cell”. Unlike the ideas discussed above, which were published in easily visible and available sources, this one has been published in a Russian journal (whose abstracts in English are, nevertheless, available in Pubmed). Therefore, there is nothing remarkable in that seven years later the ability of pp60^src^ to increase the level of glucose transporter protein in cell membranes was demonstrated in [21] independently of the above hypothesis. Later on, the significance of glucose transporters for malignancy gained general recognition [22]. Remarkable is the fact that the lines of conceptual developments that eventually became intertwined in the idea that excess glucose can drive cell populations towards increasingly cancerous states may be traced back to a single site, which is Laboratory of Endocrinology, N.N. Petrov Research Institute of Oncology, Leningrad (Saint Petersburg), USSR (Russia) as of late 1970-ies. In particular, the same journal issue where the above paper [10] by V. Dilman and M. Blagosklonny has been published contains the paper by V. Anisimov [23] where the antidiabetic biguanide buformin has been reported to increase lifespan and inhibit spontaneous carcinogenesis in rats.

The common theme of all of the above views is that increased glucose availability promotes cancer, and increased glucose consumption is a trait of cancer cells. The latter idea is actually traceable back to the Warburg effect (reviewed in [24] within a context close to the present one). However, the question remains what, if not the direct effects of some oncogenes, such as mentioned in [25], can increase the ability of cells to consume glucose? An emerging idea is that increased glucose availability is a factor of selection of cells for their ability to consume glucose [12–14], so that “clones that can take advantage of the increased supply of energy will gain the advantage” [14] over normal clones. Importantly, for effects supported by selection, even a subtle selection factor is sufficient to make such effects significant in the long run, i.e. with increasing age, by gradual accumulation of small genetic and epigenetic alterations fixed by selection because under higher glucose availability they provide more proliferative and/or survival advantages to their bearers.

### The other sides of glucose

Clearly, an indispensable prerequisite of evolution by natural selection is the variability of traits under selection. The high phenotypic instability of cancer cells and its role in cancer progression are well recognized [26–30]. Importantly, the list of possible contributions to modifications of nucleotides in DNA and amino acids in chromatin includes the ability of any reducing sugars, including glucose, to damage macromolecules by covalent modification (glycation) [14, 31–34].

An important link between increased glucose and damage to macromolecules, including DNA, is provided by the intermediates of glucose metabolism via glycolysis, the triose phosphates glyceraldehyde-3-phospate and dihydroxyacetone phosphate, which are prone to spontaneous transformation into highly toxic methylglyoxal able to damage DNA and thus to contrite to cell heterogeneity. Possible roles of methylglyoxal as a mediator between diabetes mellitus and cancer have been extensively discussed [35].

A significant aspect of macromolecular damage by glucose is modification of the extracellular matrix proteins and, thus, disruption of cell interactions with microenvironment, including those that keep premalignant cells in check [36].

Thus glucose, being an important contributor to “nonenzymatic molecular damage as a prototypic driver of aging” [37], may also contribute to somatic mutagenesis and to epigenetic variations. However, no differences between the levels of precancerous lesions were found upon comparing population where differences between cancer incidences may be attributable to differences in metabolic conditions (reviewed in [13]). Therefore, the metabolic differences may be translated into oncological disparities not via somatic mutagenesis (cancer initiation), but rather via differences in the factors, including glucose, that drive the selection of cell clones towards increasingly malignant traits (cancer promotion).

What may be a novelty in the modern views compared with the initial ideas is that the age-dependent increase in the risk of cancer is attributed to the attenuation of the systemic metabolic control of cancer [14] rather than to metabolic pushing of cells into a cancer-prone state, as it may follow from the initial physiological concept [38] and its molecular biological reincarnation [39, 40]. However, an increased availability of glucose will promote cancer either way.

With regard to body protection from cancer, the generally recognized immunological surveillance may be more important than the putative metabolic control, which is more likely to be a contributing rather than the determinative factor. The extent of the contribution may be a matter of discussion, so as the extent of the contribution of age-dependent metabolic changes to age-associated decline in the immunological surveillance. A recent study strengthens the evidence that, in humans, thymus involution is the major contributor to age-dependent immune deficits [41]. However, this does not rule out other contributors, including metabolic ones, which are more likely modifiable as compared with others. No data for making inferences as to the role of hyperglycemia in thymus involution are available so far, as well as none of the follow-up studies planned to establish how human health is associated with baseline indices of glucose tolerance (see the next section) included an evaluation of the conditions of the thymus and thymus-dependent parameters.

The ability of metabolic disturbances that hinge on impaired glucose tolerance to compromise immunity is implicit in the concept of metabolic immunodepression suggested 40 years ago [1, 15]. It is increasingly recognized that both hyperglycemia and hyperlipidemia and obesity promote inflammation (reviewed, e.g., in [36]). Although lipids seem to be more important than glucose as direct factors of inflammation and metabolic immunodepression, intimate metabolic relationships between glucose and lipids make reasons to suggest that “nobody can be immunologically fit without being metabolically fit [42].

### What is it to be metabolically fit, if not merely to be not (pre)diabetic?

Most studies of relationships between carbohydrate and/or lipid metabolism and cancer are related to Type II diabetes and/or obesity and the metabolic syndrome and unequivocally suggest that these conditions increase the risk of cancer, albeit differentially with regard to different locations (reviewed in [43–45]). Interestingly, cancer incidence is increased in Type I diabetes [43, 46–48]. Neither increased insulin and IGF signaling nor obesity can be responsible for that. In fact, the most apparent common physiological (biochemical) feature of both types of diabetes mellitus is the increased exposures of tissues to glucose. It is also worth mentioning that even non-diabetic hyperglycemia is associated with poorer prognoses for cancer patients [49].

Luckily, most living people have no overt diabetes nor clinical cancer. Can they benefit, in the oncological terms, from managing their glucose in a possibly better way? A basis for making an idea about it may be found in the meta-analysis [50] of published results of 97 prospective studies providing data about associations between death rates and baseline fasting glucose levels, among other variables. Of the 715,061 participants included in the analyses, only 40,116 (6%) had diabetes at the time of enrollment. The analyses of associations between fasting glucose and death rates was based on data related to 12.3 million person-years at risk (median time to death, 13.6 years) and 123,205 deaths, including 41,320 from cancer and 44,407 from vascular disease. The data were adjusted for baseline age, sex, smoking status and BMI.

**Figure 1:**
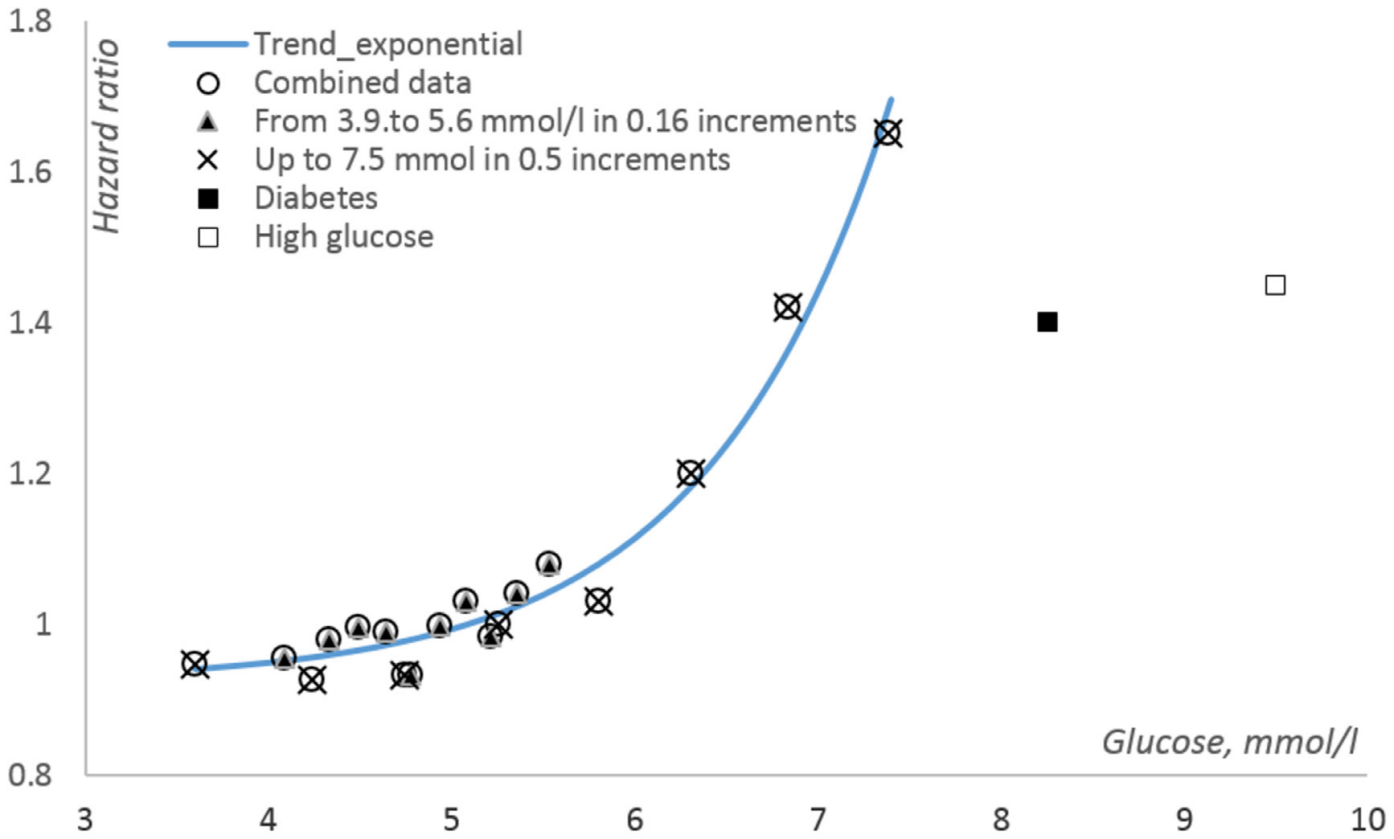
Cancer risk vs. fasting glucose according to data published in [50]. The trend line relates to non-diabetic subjects, i.e., the points (squares) related to recognized diabetics and to people with diabetic levels of fasting glucose are not accounted for. The data at the base of the index of cancer mortality, which is defined as hazard ratio in the source paper, originate from very different studies and thus had to be heavily processed in order to derive an unified integral index from them. The procedures used for this processing are beyond the scope of the present discussion, and the results of the processing are taken for granted here.

**Figure 2:**
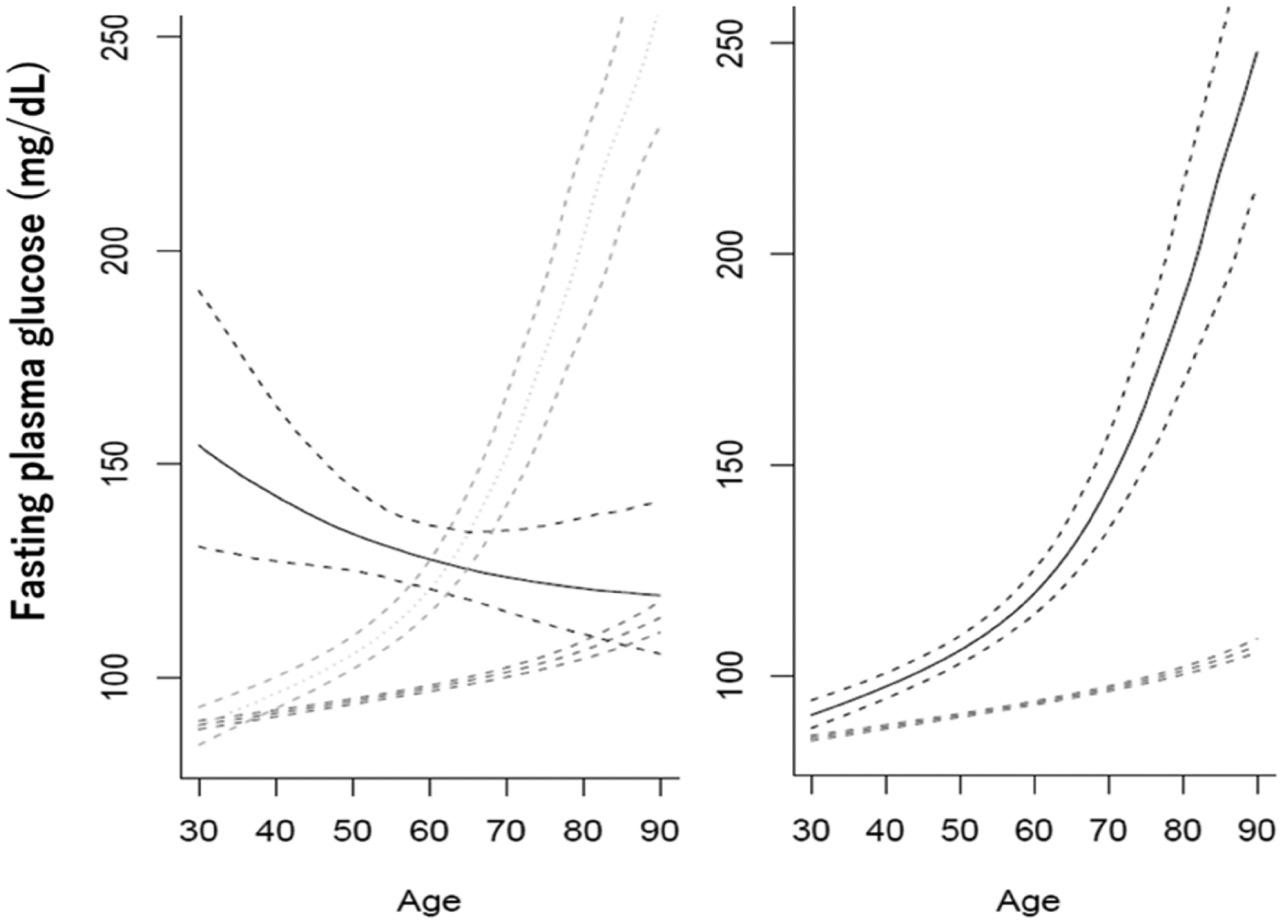
Types of trajectories of changes in fasting glucose with increasing age in men (left) and women (right). The plots are reproduced from Figures 1 and 2 of [61] with kind permission from Wiley.

Figure 1 of the present paper shows how cancer-related mortality increases with increasing fasting glucose in non-diabetic subjects. The mortality trend is derived from two figures presented in [50]. One of them (Figure 2A in [50]) shows indexes of cancer-related mortality vs. baseline fasting blood glucose in initially non-diabetic subjects having fasting glucose levels from less than 4.0 up to 7.5 mmol/l. This interval is divided in eight 0.5 mmol/l increments. The source figure contains two more points at ca. 8.25 mmol/l and 9.5 mmol/l. The first one relates to subjects defined as diabetic based on self-reports or treatment for diabetes, whereas 9.5 mmol/l is the mean fasting glucose in subjects with fasting glucose above 7.5 mmol/l (no upper limit is defined) who were not assumed as diabetic based on the two above criteria. It is unlikely however that they were not diabetic actually, their fasting glucose being so high. The other source figure (Supplementary Figure 6 in [50]) shows similar data for subjects judged as non-diabetic at baseline and having fasting glucose levels in the range from 3.9 mmol/l (70 mg/dl) to 5.6 mmol/l (100 mg/dl), which is subdivided into smaller sub-intervals than in the source Figure 2. Non-diabetes-related points derived from both source figures are consistent with a common exponential trend, which suggests that when fasting blood glucose increases from 4 to 6 mmol/l, i.e. within the range assumed as normal, the risk of death from cancer increases by 20%. Actually, many people with fasting glucose up to 7.5 mmol/l are still rated as non-diabetic. Therefore, as much as 30% of cancer risk unrelated to diabetes mellitus is modifiable by managing glucose tolerance. The reason why cancer-related death rate in diabetic subjects looks lower than in non-diabetic subjects having fasting glucose in the range from 6.5 to.7.5 mmol/l may relate to that the risk of cardiovascular deaths in diabetics is increases so much (see Figure 2B in [50]) that it effectively competes with the risk of cancer-related death. It should be noted that the points at 8.15 and 9.5 mmol/l still suggest that cancer risk upon this range of fasting glucose is higher than in subjects whose fasting glucose is below ca. 6 mmol/l.

In a more recent meta-analysis [51], which specifically addressed the relationships between the incidence of cancer (instead of cancer-associated mortality) and fasting blood glucose, cancer risk was found to be 1.32 times higher in subjects who have fasting glucose above 6.1 mmol/l vs. those having lower fasting glucose. Importantly, subgroup analysis suggested that the insulin-IGF-1 axis does not fully explain the association between glucose and cancer.

Still another meta-analysis of prospective studies revealed that cancer incidence is increased in prediabetes irrespective of which threshold fasting glucose levels were used to define this condition [52].

Figure 1 suggests that the risk of cancer increases with the level of tissue exposure to glucose in an almost exponential fashion. Therefore, an increase in cancer risk must be more apparent at higher exposure indices, such as fasting blood glucose. Indeed, upon a 9.2 ± 4.7 years follow-up of almost 302,000 subjects [53], an increase in cancer risk was found only among those who were attributed to the highest quintile of blood glucose levels (6.88 ± 1.61 mmol/l). Surprisingly, no association between fasting blood glucose and the risks of deaths attributed to other causes but cancers was apparent in these subjects, who all were non-diabetic at baseline.

Another indicator of tissue exposure to glucose, the percent of glycated hemoglobin HbA1c, was also found to correlate positively with cancer risk [54]. The most recent study [55] included 11,336 men and 18,293 women aged 46–80 years. The median follow-up duration was 8.7 years. Cancer incidence was assessed by systematic surveys. Cancer risk was adjusted for age, sex, geographic area, body mass index, smoking status, physical activity, alcohol, coffee, vegetable and total energy consumption, and history of cardiovascular disease. Cancer developed in 1,955 individuals. Higher HbA1c levels within both the non-diabetic and diabetic ranges in individuals without known diabetes were associated with higher overall cancer risk. Only the risk of liver cancer was highest in the lowest HbA1c group. After excluding liver cancer, HbA1c levels were linearly associated with the risk of all cancers.

However, in a study of 440,000 patients who were diabetic at baseline and were followed for cancer over eight years, no association between overall cancer incidence and HbA1c and blood glucose was reported [56]. A positive association was found with only pancreatic cancer, and a negative association, with prostate cancer. The authors reasoned that in the studies where diabetic, pre-diabetic, and non-diabetic patients were included, and judgments hinged on baseline measurements, higher baseline glucose could mean that the respective subjects could become diabetic in the course of follow-up, so that the increased cancer incidence or death rate could reflect the increased incidence of newly developed diabetes, which per se is associated with increased cancer risk. It may be added to the above reasoning that, in diabetic patients, the effects of diabetic complications may be more significant than the effects of glucose and thus mask them. Other causes of discrepancies between different studies may include differences in diagnostic and cut-off criteria, ethnicities, follow-up terms, statistical treatment procedures, to name a few. The discrepancies include not only the degrees of association of glucose-related indices with cancer risk but also the spectrums of cancers found in different studies.

Nevertheless, the bulk of evidence provided in the above publications and references therein, which cannot be covered here within the space possible for the present discussion, is consistent with that increased exposure of body cells and tissues to glucose is associated with increased cancer risk.

It is important in this regard that it usually takes about a decade and even more time for a premalignant lesion to develop into a clinical cancer. This means that almost all cancers and cancer-related deaths recorded in the above and similar studies represented the culminations of processes that had started long before the onset of a study. Even if a subject has become diabetic during follow-up, it only means that his/her cancer could develop under increased exposure to glucose long before he/her was even included in the study.

With regard to these temporal relationships, the question arises how glucose tolerance may change within the spans of time required for cancer to develop.

It is commonly believed that glucose tolerance decreases with aging in humans (reviewed in [57]). Recent evidence, however, suggests important caveats regarding this belief. First, period populational studies [58, 59] reveal that, even though the median estimates of fasting glucose, 2-h postprandial glucose, and HbA1c levels are higher in 60+ vs. 40–59 vs. 18–39 years groups, the differences between age groups (e.g. ca. 75 vs. 100 mg/dl fasting glucose in the youngest vs. oldest group) are much smaller than the variances of the respective indices in each of the age group (e.g., ca. 95% of fasting glucose levels in the youngest group fall within a 60 to 130 mg/dl interval), and the differences are attributed to extended right shoulders of the respective distributions, their left extremes being the same. This means that differences in the levels of exposure of body tissues to glucose established early in life may persist through the rest of life and, therefore, the baseline estimates of fasting glucose used to assess their association with cancer risk may indeed reflect the lifelong exposure of body cells to glucose, higher exposure being a factor of higher cancer risk.

At the same time, longitudinal trajectories of changes in glucose tolerance may be quite different in different subjects [60, 61]. Exemplary cases are shown in Figure 2.

In men, fasting glucose may increase from ca. 75 mg/dl at 30 years either moderately (89% of subjects) or sharply (8%), or it may decrease from initially high levels of ca. 150 mg/dl (3%). In women, moderate and sharp increase are exhibited by 94% and 6% of subjects, respectively. In any case, tissue exposure to glucose, which may be judged about based on the under-the-curve areas, is higher in those who have initially higher levels of fasting glucose or higher rates of increases in these levels.

No explanations are suggested in [61] for what may be beneath the different trajectories of age-associated changes in blood glucose. May it be that the awareness of inappropriate metabolic conditions reflected by high glucose urge men to take measures, such as diet, exercise, medication, etc.? Then why there is no case of no age-dependent decreases in initially high blood glucose levels? In another report on longitudinal changes in blood glucose, albeit over a shorter period of about 5 years, an “elevated-stable pattern” of changes in blood glucose was distinguished. “Individuals in the elevated-stable pattern were more likely to be men, have lower education, drink alcoholic beverages, use antihypertensive medications, and have concurrent cardiovascular risk factors” [60]. It is interesting, in this regard, that in men as well as in women with the sharp increases in blood glucose, increases in cardiovascular diseases were the highest, whereas in men with initially high blood glucose levels, which subsequently were decreasing, the increase in cardiovascular diseases was the lest significant [61].

May it be that the two types of increase in blood glucose are associated with either genetic differences or different attitudes towards taking care of one’s fitness? It would be interesting to compare the findings shown in Figure 2 with records concerning cancer risk in the cohort described in the source paper [61]. So far, inferences regarding this issue cannot be but speculative. Yet, the above discussion suggests that cancer incidence must be higher upon initially higher blood glucose levels anyway and must be lower, so as the risk of cardiovascular and other health problems, as far as the age-associated increase in blood glucose is moderated or even reversed with proper measures. Assuming the latter is the case, may it be that the resulting deceleration of increases in the incidences of the main age-associated diseases means that aging is decelerated by decreasing the exposure of body to glucose?

One more point of concern relates to age-specific norms of fasting glucose and related indices. One of ideas put forward by V.M. Dilman [1, 15, 19, 20, 38] was that the very notion of age-specific norm is misleading. Only those levels of, say, glucose that are associated with the minimal risks of health problems are normal. Therefore, any levels of fasting glucose out of the range of about 50 to 75 mg/dl are abnormal. No one, when he/she is 64, should deceive him/herself by thinking that he/she is metabolically fit while having, say, 90 mg/dl fasting glucose, which is conventionally believed to be normal for that age.

## Conclusions

**Figure 3:**
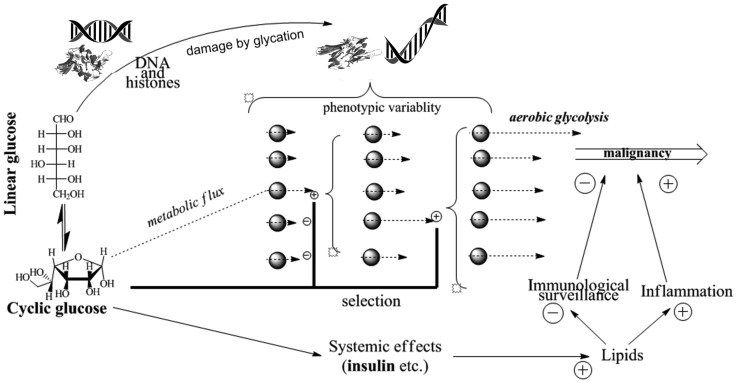
Pathways of glucose involvement in age-associated cancer promotion.

It follows from the above epidemiological evidence that exposure of body tissues to glucose is a factor of carcinogenesis. Mechanistically, this may be because glucose can affect (i) separate cells (via promoting somatic mutagenesis and epigenetic instability), (ii) cell populations (via being a factor of selection of phenotypic variants in cell populations for higher glucose consumption and, ultimately, for high aerobic glycolysis); (iii) cell microenvironment (via modification of extracellular matrix proteins), and (iv) the systemic levels (via shifting the endocrine regulation of metabolism toward increasing blood lipids and body fat, which compromise immunological surveillance and promote inflammation). Interactions between these effects of glucose are illustrated in Figure 3.

As a matter of fact, reduced glucose is a common result of several recognized complimentary means of cancer prevention and treatment, i.e. calorie restriction [62–65], increased physical activity [66–69], and antidiabetic drugs, including metformin [16–18, 70], all of which are known to increase lifespan in experimental mammals. However, only calorie restriction does it in the way that is consistent with mortality and survival patterns indicative of deceleration of aging [71].

Anyway, the newest findings and concepts strengthen the old ideas that metabolic rehabilitation of cancer patients is a potent adjuvant treatment modality and that to prevent age-dependent loss of body control over glucose is to help body to control cancer [1, 15, 19, 38], whatever theory of aging is put forward to explain this.
